# Cystatin C assay validation using the immunoturbidimetric method to evaluate the renal function of healthy dogs and dogs with acute renal injury

**DOI:** 10.14202/vetworld.2022.1595-1600

**Published:** 2022-06-30

**Authors:** Fabiola de Oliveira Paes-Leme, Eliana Matias de Souza, Mariah Gois Ceregatti, Marco Túlio Gomes Campos, Patricia Donado Vaz de Melo, Adriane Pimenta da Costa-Val

**Affiliations:** 1 Department of Clinics and Surgery, Veterinary School, Federal University of Minas Gerais, Belo Horizonte, Brazil; 2 Labtest Diagnostica SA, Lagoa Santa, Brazil.

**Keywords:** canine, immunoturbidimetry, kidney

## Abstract

**Background and Aim:**

Acute kidney injury (AKI) is associated with a grave prognosis. A clinical assessment of kidney function can be performed based on the glomerular filtration rate (GFR). Cystatin C (CysC) can indicate the GFR or kidney function and its measurement is currently performed using immunological methods such as nephelometry, immunoturbidimetry, and enzyme-linked immunosorbent assays in human medicine. However, these techniques are not specific for use in veterinary medicine. This study aimed to validate an immunoturbidimetric assay for serum CysC (sCy) in dogs, determine the sCy reference intervals for healthy dogs, evaluate sCy stability in serum samples, and compare sCy with serum creatinine (sCr) in healthy dogs and dogs with AKI.

**Materials and Methods:**

Forty-three dogs were divided into a control group (n = 19) and an AKI group (n = 24). An immunoturbidimetric method including commercially available human CysC calibrated with canine CysC was used to evaluate canine serum samples.

**Results:**

An average recovery of 97% was observed for canine serum samples. The reference interval for CysC in healthy dogs was 0.57–1.29 mg/L. The sCy concentration in dogs with AKI was significantly higher (2.82 ± 1.46 mg/L) than in healthy dogs (0.93 ± 0.18 mg/L). Statistical analysis confirmed a strong correlation between sCy and sCr (r = 0.94; p < 0.05) in dogs with AKI.

**Conclusion:**

The immunoturbidimetric method of evaluating sCy yielded satisfactory results and can be used for canine samples when a species-specific calibrator is used. Furthermore, sCy is a reliable marker of renal dysfunction in dogs. It is best to store samples for sCy evaluation at temperatures between 4°C and 8°C.

## Introduction

Acute kidney injury (AKI) is generally associated with grave outcomes, has an incidence rate of 12–63%, and is associated with mortality rates between 54.2% and 86% [1–4]. The early diagnosis of AKI is challenging because of the insufficient detection ability of the markers currently used, such as urea and creatinine [5]. Renal disease detection is essential so that renoprotective therapies can be initiated early during the disease process, thereby promoting slower progression [6]. The glomerular filtration rate (GFR) can aid in the clinical assessment of kidney function and is commonly assessed by evaluating serum creatinine (sCr). However, sCr is considered an insensitive marker for detecting an early decline in kidney function. Furthermore, muscle mass is an essential nonrenal factor that affects sCr [7]. Cystatin C (CysC) is a small protein (13 kD) used to mark GFR or kidney function and is considered superior to sCr as a marker of GFR in dogs [8]. CysC is currently measured using immunological methods such as nephelometry, immunoturbidimetry, and enzyme-linked immunosorbent assays. These techniques are quick, non-invasive, precise, and automated; therefore, they are advantageous during laboratory routines because they allow for timely results and decision-making in clinical practice. However, these techniques are not specific for use in veterinary medicine since they use human polyclonal antibodies. Assays and human kits are used for animal species in clinical practice; however, it is necessary to perform procedures that verify the accuracy and reliability of the results [9].

Previous studies [8, 10] have evaluated serum CysC (sCy); however, the methodology used during these studies and population types varied [11]. Therefore, it is necessary to continue such studies to verify the performance of CysC for the early diagnosis of AKI in dogs and compare the results to those obtained using traditional methods [8, 10–12]. Hence, it is necessary to standardize a highly sensitive marker of renal function capable of detecting early renal damage and to analyze that marker in different situations to verify its effectiveness and reliability.

This study aimed to validate the existing immunoturbidimetric method used to measure canine sCy, establish reference intervals for sCy in healthy dogs, evaluate the stability of sCy samples during storage, and investigate whether sCy levels are greater in dogs with AKI than in healthy dogs.

## Materials and Methods

### Ethical approval

The procedures were in accordance with the guidelines for animal use of the Federal University of Minas Gerais (CEUA UFMG) under protocol 56/2015 and were approved by the Ethics Committee.

### Study period and location

The study was conducted from March 2015 to March 2016 in Belo Horizonte (19° 48’ 57” S, 43° 57’ 15” W), which is the capital and largest city of Minas Gerais State and the ninth largest urban area in Brazil. The city is ranked sixth in population, with 2,530,701 inhabitants; it is ranked 20^th^ on the human development index in Brazil (0.810).

### Animals

Forty-three dogs were divided into the control group (n = 19) and AKI group (n = 24). All patients were owned by volunteers who signed an informed consent form to allow the sampling procedures and authorized the study data. Clinical data were evaluated and a clinical examination, complete blood count analysis, urinalysis, and chemical evaluation were performed. Clinical evaluation results, serum biochemistry results, and ultrasound results were used to classify patients in the AKI group in accordance with the staging proposed by the International Renal Interest Society [13]. For all analyses, dogs were considered normovolemic and were fasted a maximum of 12 h before blood collection.

### Sampling and tests procedures

Blood samples (5 mL) were collected by venipuncture of the external jugular vein in Ethylenediaminetetraacetic acid 10% for the complete blood count analysis and in serum for the serum biochemistry analysis; CysC determination was performed using Cobas Mira Plus Roche® based on PETIA methodology and Cistatina C Turbiquest Plus^®^ (Labtest, Lagoa Santa, Brazil) reagents. The sCr value was determined using an enzymatic method and a commercial kit (Kovalent, Rio de Janeiro, Brazil). The sCr concentration is considered the gold standard for characterizing normal (<1.4 mg/dL) and altered (>1.4 mg/dL) renal function in dogs. An analysis was performed to determine the behavior of sCy.

Two canine serum samples and a normal sample of canine sCy (CysC canine *Escherichia coli*; RD472009100) with a concentration of 0.1 mg/L were used for the validation tests. Canine CysC values were measured in canine serum samples containing low (1.48 mg/L) and high (5.69 mg/L) concentrations of this analyte, which were used to determine the precision, accuracy, quantification limit, and stability.

### CysC assay validation

The validation procedures were performed in compliance with the requirements of the validation process and the standards of the Brazilian regulatory agencies [14, 15]. During all tests, the mean, standard deviation (SD) and coefficient of variation (CV) were calculated. Calibration, linearity, precision, quantification limit, accuracy, prozone effect, and sample stability were established.

### Calibration

The calibration curve was performed using the canine antigen (CysC canine *E. coli*; RD472009100; 0.1 mg). The points on the curve were as follows: Point 0, 0 mg/L (saline 0.9%); point 1, 0.62 mg/L; point 2, 1.25 mg/L; point 3, 2.5 mg/L; point 4, 5.0 mg/L; and point 5, 10.0 mg/L.

### Precision

The intra-assay precision (repeatability) of the immunoturbidimetric method using CysC for dogs was evaluated by calculating the mean, SD, and CV of the results obtained from two serum samples: One contained a high concentration of CysC (5.69 mg/L) and the other contained a low concentration of CysC (1.48 mg/L). Five measurements were performed in triplicate for each sample on the same day, resulting in a total of 30 measurements; deviations >15% were not allowed [14, 15]. The intermediate precision of this method was evaluated by calculating the mean, SD, and CV of the results obtained from the refrigerated serum samples containing high values (5.69 mg/L) and low values (1.48 mg/L) of canine CysC by performing measurements in triplicate for each sample on 3 different days, resulting in a total of 18 dosages.

### Recovery percentage

To evaluate the recovery percentage, the difference in the obtained mean and the supposed concentration was measured.

### Linearity and detection limit

To determine the linearity of sCy detection, the purified canine antigen (CysC canine *E. coli*; RD472009100; 0.1 mg) was first prepared to achieve 100 mg/dL; then, it was diluted (saline 0.9%) to 1:2, 1:8, 1:16, 1:32, 1:50, and 1:100. The curve was constructed according to the manufacturer’s instructions after calibration. The detection limit was evaluated using the measurements of the standard serum sample diluted to the lowest value of the analytical assay range.

### Hook effect

The hook zone effect was verified using a device calibrated to the maximum reading value (10 mg/L). Analyses were performed in triplicate using standard serum (CysC canine *E. coli*) with canine CysC quantities of 10, 12, and 25 mg/L.

### Sample stability

For the stability analysis, samples were divided into different Eppendorf tubes, and each was used at different analysis times (12 h and 7 days). Samples were kept at room temperature (25–28°C), in the refrigerator (4–8°C), and frozen (−20°C).

### Reference intervals

The reference intervals adopted for this study were based on measurements of sCy in 19 healthy dogs that comprised the control group. To obtain CysC reference intervals, quintiles 0.025 and 0.0975 were used to determine the mean ± SD.

### Correlations of sCy and sCr concentrations for the diagnosis of AKI

To establish the correlation between sCy and sCr in dogs with AKI, 24 dogs with AKI (sCr > 1.4 mg/dL) were divided into three groups according to the degree of AKI: Group 1, sCr between 1.7 and 3.0 mg/dL (n = 13); Group 2, sCr between 3.1 and 5.0 mg/dL (n = 5); and Group 3, sCr > 5.0 mg/dL (n = 6).

### Statistical analysis

All variables were analyzed descriptively. Quantitative variables were analyzed using the SD, whereas qualitative variables were analyzed using absolute and relative frequencies (%). The Kruskal–Wallis test and Spearman’s correlation were applied to non-parametric variables. The results were considered significant at p < 0.05.

## Results

### Calibration

The results obtained by an analysis of different concentrations of canine sCy (CysC canine *E. coli*; RD472009100; 0.1 mg/L) resulted in a calibration curve that was considered adequate when the values measured were proportional to the known calibrated concentration. For the CysC calibrated sample with a concentration of 0.62 mg/L, the mean was 0.61 mg/L (SD, ± 0.09 mg/L) and the CV was 12% after three measurements. For the calibrated sample with a concentration of 1.25 mg/L, the mean was 1.46 mg/L (SD, ± 0.10 mg/L) and the CV was 6.8% after three measurements. For the calibrated sample with a concentration of 2.5 mg/L, the mean was 2.51 mg/L (SD, ± 0.17 mg/L) and the CV was 6.9% after three measurements. For the calibrated sample with a concentration of 1.25 mg/L, the mean was 1.26 mg/L (SD, ± 0.10 mg/L) and the CV was 6.8% after three measurements. After three measurements, a mean of 4.89 mg/L (SD, ± 0.18 mg/L) and CV of 3.7% were obtained for the calibrated sample with a concentration of 5.00 mg/L. For the calibrated sample with a concentration of 10.0 mg/L, the mean was 9.78 mg/L (SD, ± 0.10 mg/L) and the CV was 2.9% after three measurements.

### Precision

The repeatability (inter-assay precision) evaluation indicated that the sample with a high CysC concentration had a CV of 5.3%; for the sample with a low concentration, the CV was 8.5%. Intermediate precision was observed when the sample with a high CysC concentration value had a CV of 3.7% and when the sample with a low CysC concentration had a CV of 7.0% (Table-1).

**Table 1:** Means and standard deviations of sCy in canine samples with high and low concentrations used to evaluate the precision of the intra-assay and inter-assay.

Canine Cystatin C (mg/L)				

Precision	Intra-assay (Repeatability)		Inter-assay (Intermediary Precision)*	
	
	High Concentration	Low Concentration	High Concentration	Low Concentration
Sample	5.69	1.48	5.69	1.48
Mean ± SD	5.13 ± 0.67	1.65 ± 0.25	5.53 ± 0.21	1.46 ± 0.10
CV (%)*	5.3	8.5	3.7	7.0
Measurements, n	15	15	9	9

sCy=Serum cystatin C, SD=Standard deviation, CV=Coefficient of variation.

* Evaluations were performed at intervals of 24 h, 72 h, and 7 days

### Recovery

The canine serum sample with an initial concentration of 1.48 mg/L (low concentration) presented a mean of 1.46 mg/L, thereby resulting in a 98% recovery rate. The canine serum sample with an initial concentration of 5.53 mg/L (high concentration) presented a mean of 5.53 mg/L, thereby resulting in a 97% recovery rate.

### Detection limit

For dosages with higher dilutions, a CV of 12.8% was observed.

### Hook effect

For samples with a concentration of 10 mg/L, the reading was accurate. Higher concentrations resulted in a proportional reading of >10 mg/L. There was no hook effect in samples containing up to 25 mg/L of canine CysC.

### Sample stability

Samples kept at room temperature showed a loss of up to 40% after 12 h. The samples frozen at −20°C for 7 days showed a loss rate of approximately 30%. The samples refrigerated for 7 days showed a loss rate of 15% compared to the initial sCy concentration.

### Reference intervals

The sCr concentrations ranged from 0.38 mg/dL to 1.44 mg/dL (mean: 0.82 mg/dL; SD: 0.29 mg/dL) in the control group, which had a CysC concentration range of 0.63–1.19 mg/L (mean: 0.93 mg/L; SD: 0.18) (Table-2). The sCy reference values of healthy dogs ranged from 0.57 mg/L to 1.29 mg/L. There was no correlation between sCr and sCy in the control group (p > 0.05). There was also no significant correlation between sCy concentrations and sex, age, or weight (Table-3).

**Table 2:** Means, standard deviations, and correlations between sCr and sCy of dogs in the control group and in the AKI group.

Group	sCr (mg/dL)	sCy (mg/L)	Correlation
Control (n=19)	0.82 ± 0.29^a^	0.92 ± 0.18^a^	r = 0.14 (p > 0.05)
AKI* (n=24)	3.98 ± 2.69^b^	2.82 ± 1.46^c^	r = 0.94 (p < 0.0001)

AKI=Acute kidney injury, sCr=Serum creatinine, sCy=Serum cystatin C.

* Numbers with different letters indicate a statistically significant difference (p < 0.05, Kruskal Wallis test). Reference values: sCr < 1.6 (mg/dL)

**Table 3:** Spearman correlations among sCy, body weight, age, and sex of the control animals (n = 19).

Spearman Correlation		

Characteristics	Cystatin C	p-value
Weight	r = 0.26	0.2538
Age	r = −0.07	0.7719
Sex	r = 0.17	0.4484
sCr	r = 0.14	0.5579

sCr=Serum creatinine, sCy=Serum cystatin C

### Correlations between sCy and sCr concentrations in the diagnosis of AKI

The mean values and SDs of sCr and sCy were 3.98 ± 2.69 mg/L and 2.82 ± 1.46 mg/L, respectively, in the AKI group (Table-4). There was a significant and robust correlation between sCr and sCy (r = 0.94) in animals with AKI.

**Table 4:** Means and standard deviations of sCr and sCy of healthy dogs with AKI.

Group (n)	sCr (mg/dL)	sCy (mg/L)
Control (19)	0.82 ± 0.29^aD^	0.92 ± 0.18^aD^
AKI Group 1 (13)	2.40 ± 0.40^aC^	1.76 ± 0.42^aC^
AKI Group 2 (5)	3.70 ± 0.45^aB^	2.99 ± 0.43^aB^
AKI Group 3 (6)	8.10 ± 2.0^aA^	5.02 ± 0.72^aA^

AKI=Acute kidney injury, sCr=Serum creatinine, sCy=Serum Cystatin C. Uppercase letters are used to compare columns and lowercase letters are used to compare lines. Values with different letters indicate a statistically significant difference (p < 0.05). Group 1: sCr between 1.7 and 3.0 mg/dL. Group 2: sCr between 3.1 and 5.0 mg/dL. Group 3: sCr > 5.0 mg/dL. Reference values: sCr < 1.6 mg/dL and sCy < 1.29 mg/L

### Performance of CysC as a biomarker of renal injury in animals with different degrees of AKI

In the control group (n = 19), the mean sCr concentration was 0.82 mg/dL (SD, ± 0.29 mg/dL). In Group 1, the mean was 2.4 mg/dL (SD, ± 0.40 mg/dL). In Group 2, the mean was 3.7 mg/dL (SD, ± 0.45 mg dL). In Group 3, the mean was 8.1 mg/dL (SD, ± 2.0 mg/dL). The mean sCy concentrations in the control group, Group 1, Group 2, and Group 3 were 0.92 mg/L (SD, ± 0.18 mg/L), 1.76 mg/L (SD, ± 0.42 mg/L), 2.99 mg/L (SD, ± 0.43 mg/L), and 5.02 mg/L (SD, ± 0.72 mg/L), respectively. Both markers showed mean values greater than the reference values established for the control group, regardless of the degree of renal dysfunction (Table-4).

## Discussion

The limit of detection of sCy for the present reagent and biochemical analyzer must be 0.62 mg/L. There was no prozone effect in samples with canine sCy concentrations up to 25 mg/L, although the existence of serum samples with such high concentrations is unlikely.

Samples were frozen at −20°C or kept at room temperature for 12 h or more should not be used to evaluate canine CysC. The previous studies [10–12] used different methods of storing dog serum samples, such as frozen at −20–55°C and frozen at −80°C for more than 9 months. All of these studies considered the samples acceptable for analyses; however, time and temperature were not compared, and the percentage of recovery with regard to the initial concentration was not evaluated [10, 12, 16–18]. In the present study, refrigeration (4–8°C) was the best conservation method because the reduction rate was 15% compared to the initial concentration. For samples kept at room temperature for 12 h and frozen for 7 days, the reduction rates were 40% and 30%, respectively. Therefore, there was no significant loss of immunoreactivity or significant degradation during refrigeration for 7 days [19].

The variation in reference intervals in the literature is evident; this might be explained by the different reagents and methods used. The immunoturbidimetric assay can result in average values ranging from 0.18 to 1.60 mg/L [10, 16–18, 20, 21]. Our results were similar to those mentioned, and the obtained reference value ranged from 0.57 mg/L to 1.29 mg/L. It was also observed that the distribution of CysC followed a Gaussian distribution; therefore, the probability that the evaluated population had a sCy concentration within this range was 95%. However, to determine the reference values for sCy, studies with more individuals should be performed in different regions using the same analytical procedure [22]. Because of the different analytical methods, calibration, antisera, and age distribution, it is not easy to compare the results of different studies, thereby demonstrating the importance of standardizing the methodology [12]. Normalizing the reference values for sCy may be required in some cases for use in clinical practice [23], and the variation of these values is a great disadvantage to their routine use; this is also true for veterinary practice.

The main advantage of sCy compared to sCr is that sCy is independent of the body muscle mass; therefore, muscle loss attributable to paralysis, immobility, anorexia, malnutrition, and aging does not affect its values during the evaluation of renal function and changes little with age, sex, and diet [24]. There were no significant correlations among sCy, age, sex, or weight of the animals evaluated. In a population of 179 dogs divided into groups of 89 puppies (< 1 year), 39 adult dogs (1–8 years), and 51 elderly dogs (8–16 years), despite the lower CysC values in young and middle-aged adult dogs compared to those of puppies and elderly dogs, there was an overlap of results of the different ages evaluated; because the difference was moderate, the same reference values could be used regardless of age [10]. Animals that weighed 5 kg had lower values than dogs that weighed more than 5 kg [12], and greater sCy concentrations were observed in older animals [20]. Therefore, the contradictory results in the literature highlight the need for additional studies, including more animals [8].

The sCy levels were significantly higher in dogs with renal dysfunction. In addition, there was a significant and robust correlation between sCy and sCr under these conditions (r = 0.94). sCy is a good marker of GFR in dogs and humans with AKI, which is in agreement with the findings of previous studies involving dogs with chronic kidney disease [10, 12, 20, 25]. However, during a previous study [10], sCy did not present good sensitivity for diagnosing AKI in dogs because only one dog with AKI had a CysC value above the reference interval. Therefore, it needs to be considered that the experimental model used by that study did not precisely reflect AKI.

sCy showed excellent performance when used for dogs with different degrees of kidney damage. However, the individualized analysis of the animals demonstrated less sensitivity of this biomarker to detect renal damage in animals with a mild loss of renal function. Two animals in Group 1 still had sCy concentrations within the reference interval (< 1.29 mg/L) despite renal injury, which was detected by sCr (1.7–3.0 mg/dL). This suggests the lower sensitivity of sCy compared to sCr when evaluating animals with AKI during the initial phase [11, 26]. However, no comparison was made between sCy and sCr using the GFR; therefore, we cannot confirm the inferiority of CysC. The controversial results presented in the literature and the lower sensitivity of CysC in dogs with a mild loss of renal function reflect the need to evaluate the use of this biomarker for dogs with AKI. Most data in the literature refer to animals with chronic kidney disease diagnosed by a punctual analysis.

## Conclusion

Under the conditions of this experiment, it was determined that the immunoturbidimetric technique using human antibodies was efficient for creating the dose of canine CysC in serum samples if it is calibrated with purified canine CysC. This procedure is in accordance with the recommended criteria for the validation of laboratory methods and results in adequate precision, accuracy, and recovery. Therefore, its implementation in the laboratory routine is feasible. Serum samples should be stored at temperatures between 4°C and 8°C. Furthermore, CysC is a reliable marker of acute renal dysfunction in dogs, is not influenced by sex, age, and weight, and is significantly and robustly correlated with sCr. Therefore, it can be a reliable alternative for diagnosing and monitoring animals.

## Authors’ Contributions

EMS, FOP, and MHLA: Conceived and designed the study. EMS: Collected samples. FOP: Supervised the study. MGC, MTGC, and PDVM: Performed the laboratory procedures. EMS, FOP, MGC, and APC: Analyzed the data and edited the final manuscript. All authors have read and approved the final manuscript.
